# Arterial stroke in a child with Seckel syndrome with a pattern of non‐moyamoya vasculopathy

**DOI:** 10.1002/ccr3.8871

**Published:** 2024-05-07

**Authors:** Samin Alavi, Mitra Khalili, Paniz Mirmoghaddam, Sharareh Kamfar, Negar Shams

**Affiliations:** ^1^ Pediatric Congenital Hematologic Disorders Research Center Research Institute for Children's Health, Shahid Beheshti University of Medical Sciences Tehran Iran; ^2^ Pediatric Radiology Department Shahid Beheshti University of Medical Sciences Tehran Iran

**Keywords:** CNS vasculopathy, microcephalic primordial dwarfism, moyamoya disease, non‐moyamoya vessel network, Seckel syndrome, stroke

## Abstract

Seckel syndrome is a rare autosomal recessive disorder, characterized by growth retardation and multiple anomalies associated with CNS vasculopathy. We describe a child with Seckel syndrome who developed a stroke due to non‐moyamoya vasculopathy.

## 1 INTRODUCTION

Seckel syndrome (SS) is a rare, autosomal recessive, heterogeneous type of primordial dwarfism first described by Seckel in 1960.^1^ It almost affects one out of 10,000 children characterized by severe intrauterine and postnatal growth retardation, dysmorphic features, microcephaly, mental retardation, and beak‐like protrusion of the midface (bird headed).^2^, ^3^ The above diagnostic criteria were defined by Majewski and Goeke after reviewing 60 patients with similar manifestations.^4^ In addition, skeletal, cardiovascular, hematologic, endocrine, gastrointestinal, and central nervous system abnormalities are well recognized in patients with SS.^3^

The overall rate of central nervous system (CNS) vasculopathy in SS patients is reported to be 3.16%, with mean age of 13.5 years (6–19 years) and equal gender distribution.^5^ Other cerebrovascular lesions reported in SS include multiple intracranial aneurysms,^6^, ^7^ subarachnoid hemorrhage,^7^ moyamoya‐like^8^ and moyamoya disease (MMD).^9^ Here, we report an 8‐year‐old girl with SS who developed arterial stroke presenting with headache, facial palsy, and hemiplegia.

## 2 CASE PRESENTATION

### 2.1 Patient information

An 8‐year‐old girl who was a known case of Seckel syndrome at 2 years of age presented to the emergency department with facial asymmetry, swallowing difficulty, and left‐sided hemiplegia. The parents complained of a recent history of headache, dragging foot, and falling about 6 days earlier. She was visited in emergency department of another hospital at that time and underwent brain CT‐scan which was not conclusive. She was discharged and was recommended to have follow‐up in a tertiary specialized center.

### 2.2 Clinical findings

**FIGURE 1:**
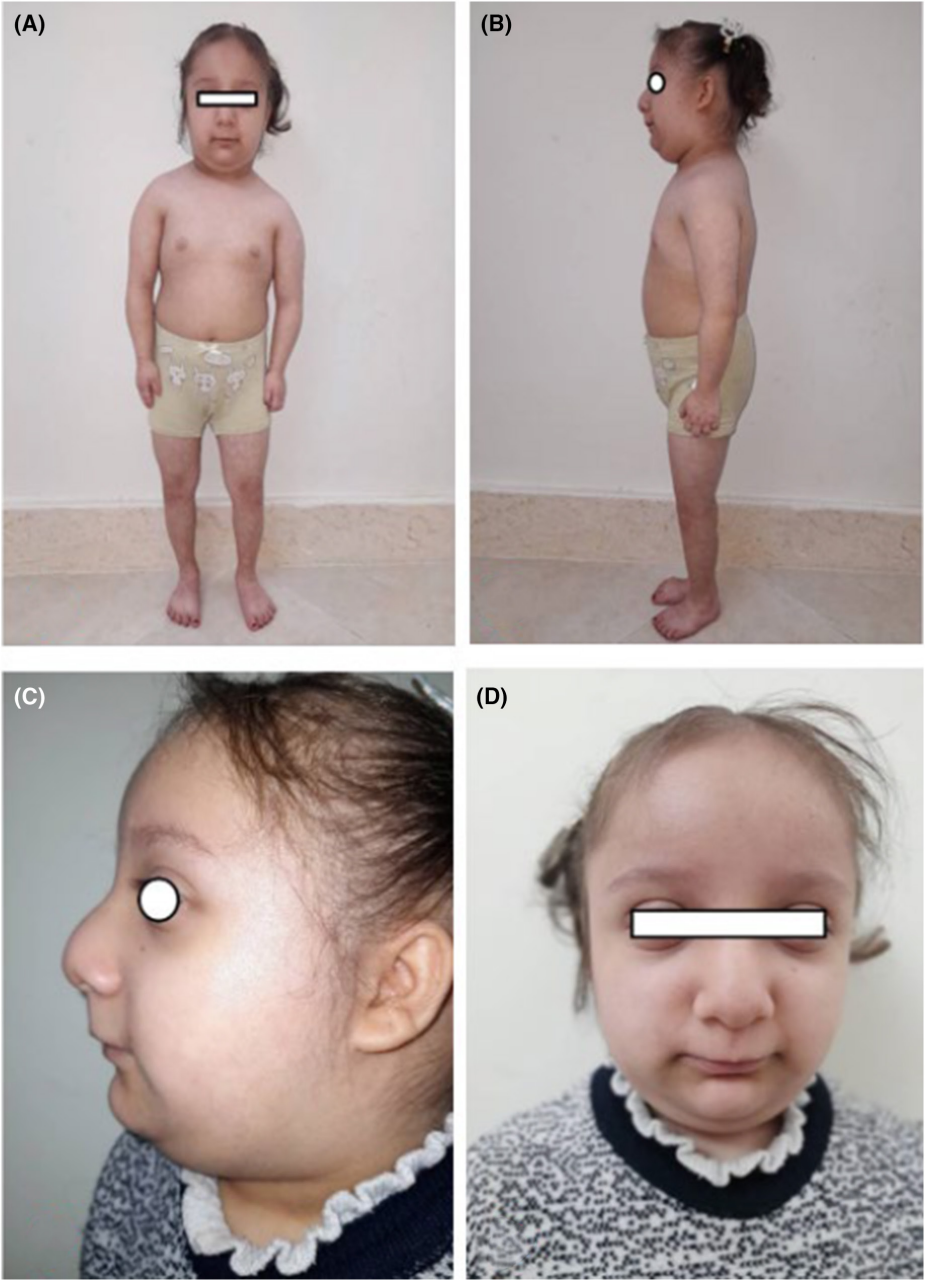
(A–D) Severe dwarfism with dysmorphic features and beaked nose, low‐set ears, prominent eyes, micrognathia, and sparse brittle hair.

Physical examination upon arrival was remarkable for severe dwarfism for a child at the age of eight with body weight of 13 kg and height of 80 cm. Psychomotor and mental retardation along with speech disability was obvious in the child. Her face was dysmorphic with a protuberant beaked nose, low‐set ears, prominent eyes, micrognathia, and sparse brittle hair (Figure 1A–D). The patient was admitted in ICU due to hemiplegia with the impression of stroke. Neurologic examination revealed decreased muscle power on the left side in both upper and lower extremities. No nystagmus was noted. She demonstrated left side upper motor neuron facial palsy.

### 2.3 Diagnostic assessment

**FIGURE 2:**
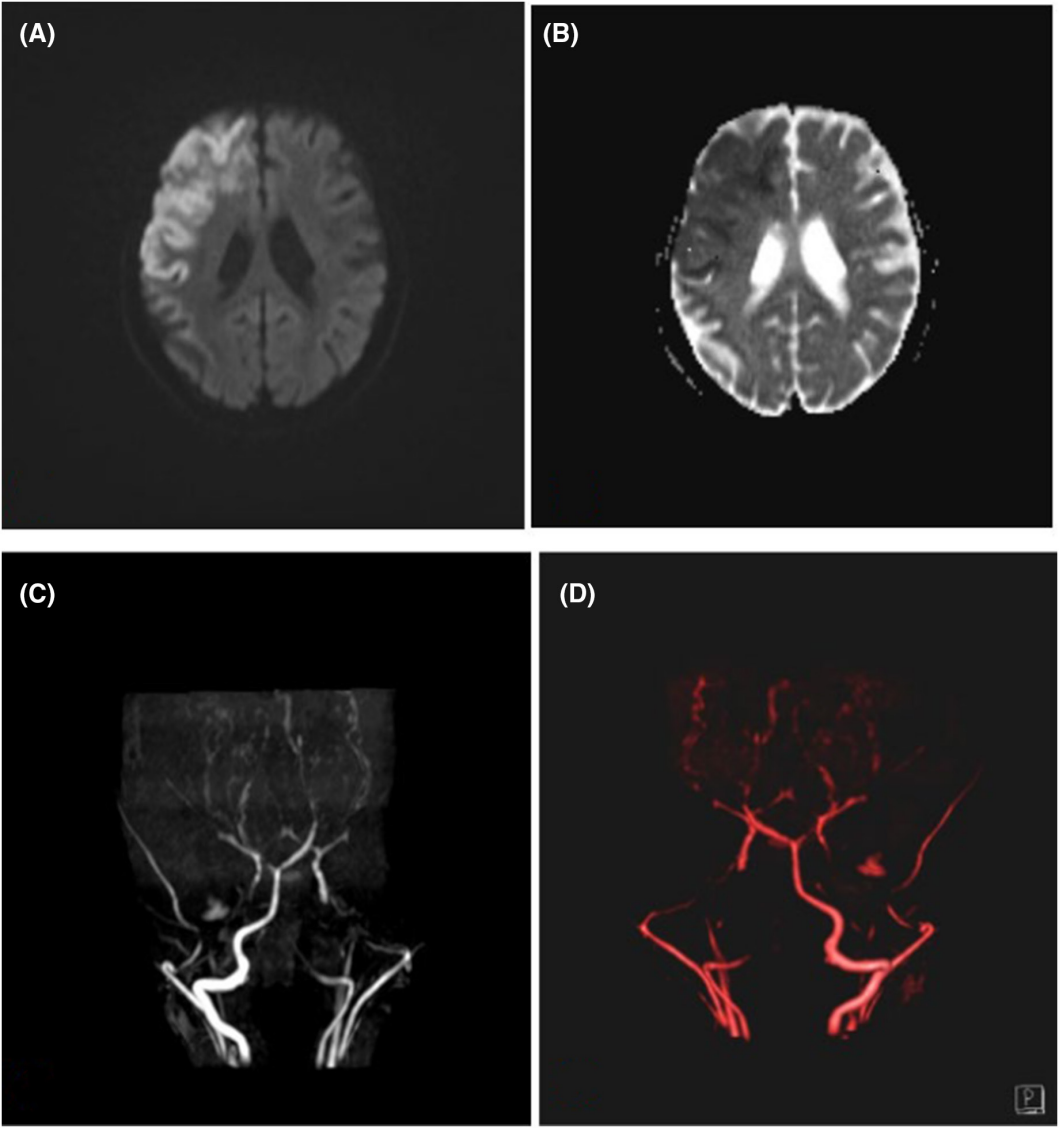
(A, B) Axial MRI images showed a bright area on diffusion weighted images (DWI) with low signals on apparent diffusion coefficient (ADC) maps in favor of acute infarction in territory of right middle cerebral artery. (C, D) Reconstruction images of brain MRA showed extreme narrowing of bilateral long segment of intracranial internal carotid arteries (ICAs), obliteration of the right lacerum segment and bilateral horizontal segment of ICAs with formation of distal non‐moyamoya collateral vessels.

According to clinical manifestations and physical examination, brain MRI was scheduled that showed a bright area on diffusion weighted images (DWI) with low signals on apparent diffusion coefficient (ADC) in favor of acute infarction in territory of right middle cerebral artery (Figure 2A,B). Reconstruction images of brain MRA showed extreme narrowing of bilateral long segment of intracranial internal carotid arteries (ICAs), obliteration of the right lacerum segment, and bilateral horizontal segment of ICAs with formation of distal non‐moyamoya collateral vessels (Figure 2C,D).

### 2.4 Diagnosis

According to her imaging and following consultation with pediatric neurologists, she was diagnosed with moyamoya‐like vasculopathy.

### 2.5 Therapeutic interventions

Although there is no definite treatment for moyamoya‐like syndromes, Aspirin 5 mg/kg was started for the patient.

### 2.6 Follow‐up and outcome of interventions

We did not perform any revascularization procedure for the patient due to lack of consent of the parents. The patient was scheduled to continue aspirin to prevent further stroke attacks.

There was a very slow course of recovery during the hospital admission. She started receiving physiotherapy and rehabilitation therapies following discharge from the hospital after a week. Ten months following the stroke, there is still some degrees of weakness in the left‐sided extremities.

## 3 DISCUSSION

The present case developed arterial stroke in the territory of right middle cerebral artery due to extreme narrowing of bilateral long segment of intracranial internal carotid arteries (ICAs), obliteration of the right lacerum segment, and bilateral horizontal segment of ICAs with formation of distal non‐moyamoya collateral vessels.

A number of CNS vascular anomalies have been reported in different types of microcephalic primordial dwarfisms (MPDs). Dilation of the CNS arteries described as aneurysms and MMD followed by their rupture, CNS hemorrhage, and strokes are among life‐threatening events observed in patients with primordial dwarfism type II; Majewski osteodysplastic primordial dwarfism type II.^10^ In a systematic review, 253 cases of SS have been identified that eight cases developed CNS vasculopathy.^5^ The overall rate of CNS vasculopathy in SS patients was reported to be 3.16% (*n* = 8/253), where MMD accounted for 1.97% of the cases. The mean age of the patients was 13.5 years (6–19 years), with equal gender distribution. The most common presenting symptoms were headache and seizure followed by weakness or coma. Aneurysms were mostly located in the basilar, middle cerebral, and internal carotid artery, respectively. The first symptom in the present case was headache followed by inability to walk and falling which proceeded to left‐sided hemiplegia.

Few reports of CNS pathologies such as intracranial aneurysms, severe hypertension resulting in cerebrovascular accidents, agenesis of the corpus callosum, cortical dysplasia, and MMD have been reported.^5^, ^8^, ^11^, ^12^ There is a report of an 18‐year‐old female patient with SS who was referred for spontaneous subarachnoid hemorrhage. Brain MRA and cerebral angiography showed bilateral narrowing of extracranial and intracranial ICAs, obliteration of the right supraclinoid segment of ICA without moyamoya collaterals, and multiple bilateral saccular aneurysms on the hypertrophied posterior cerebral arteries.^8^ Likewise, a 3.5‐year‐old boy with MOPDII has been reported who died at 8 years of age as a result of an intracranial hemorrhage due to a cerebral aneurysm associated with the moyamoya malformation.^13^ A 16‐year‐old girl with SS and moyamoya syndrome has been reported in the literature as the first case of such an association. The patient had presented with persistent headaches and a 2‐year history of hand, arm, and face numbness. Imaging studies revealed multiple completed cerebral infarcts, ischemic changes, and vascular anatomy consistent with moyamoya syndrome. This case described the potential for rapid progression of aneurysm development in this population.^9^ Previous reports have noted the frequent presence of a variety of parenchymal CNS abnormalities in patients with SS including corpus callosum agenesis, hypoplasia of the cerebellar vermis, cortical dysplasia, and pachygyria.^9^ A 6‐year‐old girl has been reported to be admitted with chief complaints of left side weakness, left‐sided facial droop, and inability to walk. MRA of the patient revealed absence of right middle and left anterior cerebral arteries with some collateral formation above the level of both ICAs which was suggestive of MMD.^14^ Reconstructed CT angiography of the cerebral arteries showed marked narrowing in the C2 and C3 segments of both ICAs, especially in the left side associated with severe short segment narrowing in the A1 segment of left ACA and focal stenosis in supraclinoid portion of right ICA.

As it is mentioned, we did not perform any revascularization procedure for the patient due to lack of consent of the parents. The patient was scheduled to continue aspirin to prevent further stroke attacks.

## 4 CONCLUSION

Screening for cerebral vasculopathy in patients with SS seems reasonable especially in patients presenting with stroke. Early neuroimaging in the screening of children with Seckel syndrome and other forms of microencephalic primordial dwarfism is highly recommended. Since cerebral vascular angiopathy is the major cause of stroke in these patients, early diagnosis and prompt management strategies including medical treatment and surgical interventions such as cerebral revascularization are critical; however, further data are required to identify the most appropriate management plan in these cases.

## AUTHOR CONTRIBUTIONS

**Samin Alavi:** Conceptualization; writing – review and editing. **Mitra Khalili:** Investigation; methodology; project administration; writing – review and editing. **Paniz Mirmoghaddam:** Resources; software; visualization; writing – original draft. **Sharareh Kamfar:** Data curation; supervision; validation. **Negar Shams:** Methodology; writing – original draft.

## FUNDING INFORMATION

There was no funding for this article.

## CONFLICT OF INTEREST STATEMENT

The authors state that they have no conflict of interest.

## ETHICS STATEMENT

Permission for publication of this case report was taken from Board of Faculty Members of Pediatrics, Shahid Beheshti University of Medical Sciences, Tehran, Iran.

## CONSENT

Written informed consent was obtained from the patient's parents to publish this report in accordance with the journal's patient consent policy.

## Data Availability

The data that support the findings of this study are available from the corresponding author upon reasonable request.
